# Life and Trial of Dr. Abner Baker

**Published:** 1846-01

**Authors:** 


### LIFE AND TRIAL OF DR. ABNER BAKER.

This is a volume of 160 pages, by C. W. Crozier, containing the circumstances of the trial and execution of Dr. Baker, to which we have already referred in this number at considerable length. We allude to the subject again for the purpose of informing our readers that the volume has been published, and that it contains much matter which we were unable to embrace in our article. Much of it is of a character which one must regret to see circulated through all the walks of society; but it is so mingled with the history of the deplorable case that it could not have been omitted. The case is obliged to assume great importance in its relation to medical jurisprudence, and as such ought to be carefully studied by every physician and lawyer. Y.

